# Supplementary material for: Smooth muscle cell Piezo1 depletion results in impaired contractile properties in murine small bowel
Source: Commun Biol. 2025 Mar 17;8:448. doi: 10.1038/s42003-025-07697-6 (PMC11914552; doi:10.1038/s42003-025-07697-6)
Supplement: Supplementary file 1 — Supplementary Information [file 42003_2025_7697_MOESM1_ESM.pdf]

Supplementary Figures

Supplementary Figure 1: Inducible depletion of Piezo1 in SMCs.

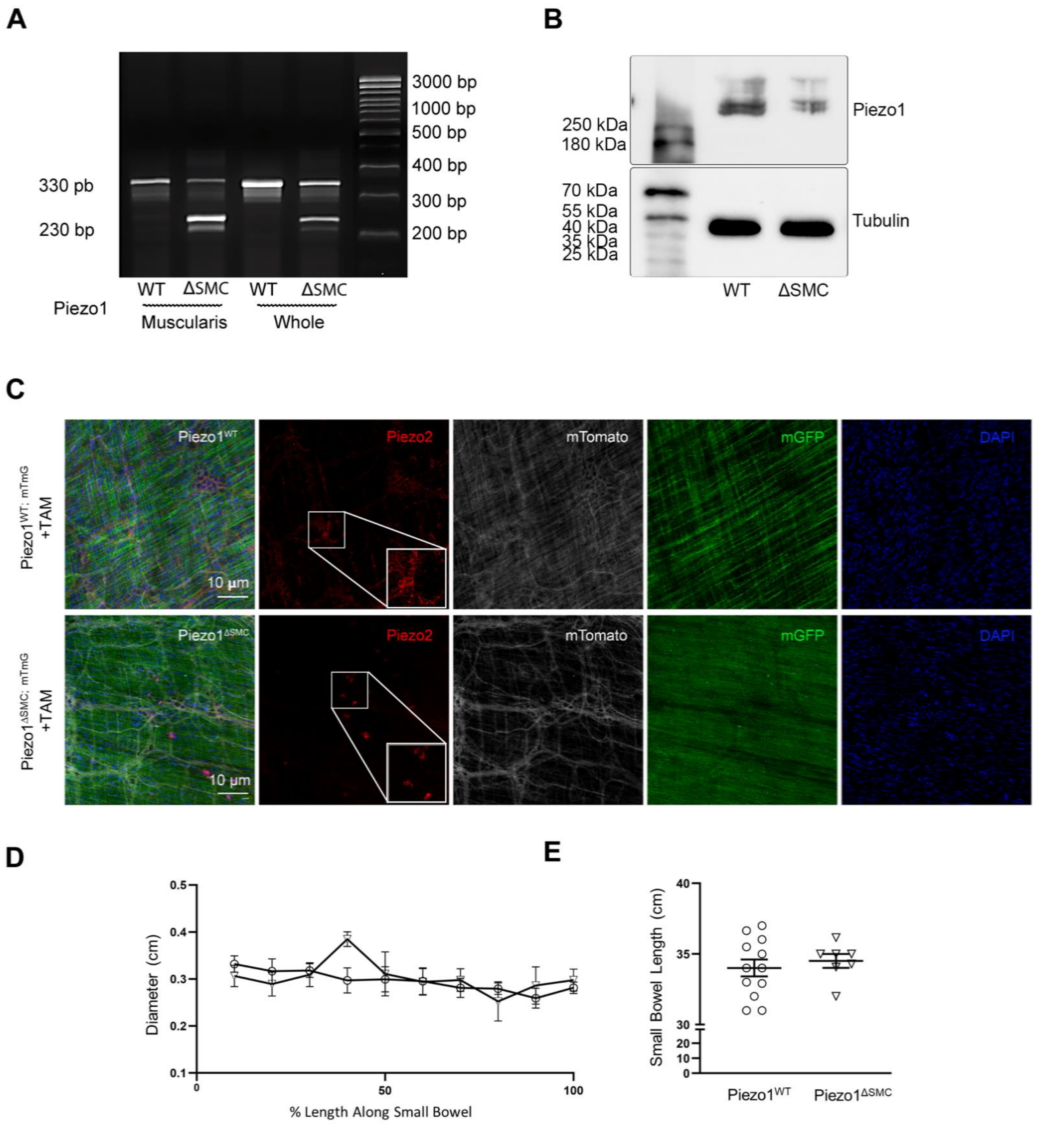

(A) *In vivo* samples from extracted muscularis or whole (all layers) of distal small bowel from Piezo1$^{\Delta SMC}$ mice have proper cleavage by 10d post-Tam treatment compared to Piezo1^{WT} mice as assessed by PCR of established genomic DNA[26]; **(B)** Muscularis samples were evaluated by Western Blot analysis using anti-Piezo1 (Proteintech) and b-Tubulin (Abcam), confirming a reduction of Piezo1 protein level in Piezo1$^{\Delta SMC}$ compared to control mice. **(C)** Immunofluorescent imaging with an anti-Piezo2 antibody (Alamone Labs; red) in WM samples from Piezo1$^{WT;mTmG}$ and Piezo1$^{\Delta SMC;mTmG}$ post-Tam treated mice reveals that Piezo2 is not located in SMC. All scale bars, 10μm. **(D)** Gross measurements of diameter measurements throughout the entire length of the small bowel divided into 10 portions of 6- to 8-week-old Piezo1^{WT} and Piezo1$^{\Delta SMC}$ mice at 10d post-Tam. (**E**) Small bowel length from the most proximal portion of the duodenum to the terminal ileum immediately before the cecum of Piezo1^{WT} and Piezo1$^{\Delta SMC}$ in 6-to 8-week-old mice at 10d post-Tam.

Supplementary Figure 2: Verification of Myh11-Cre-induced cleavage of pup IMC in an *in vitro* co-culture system and human IMCs derived from the fetal small intestine.

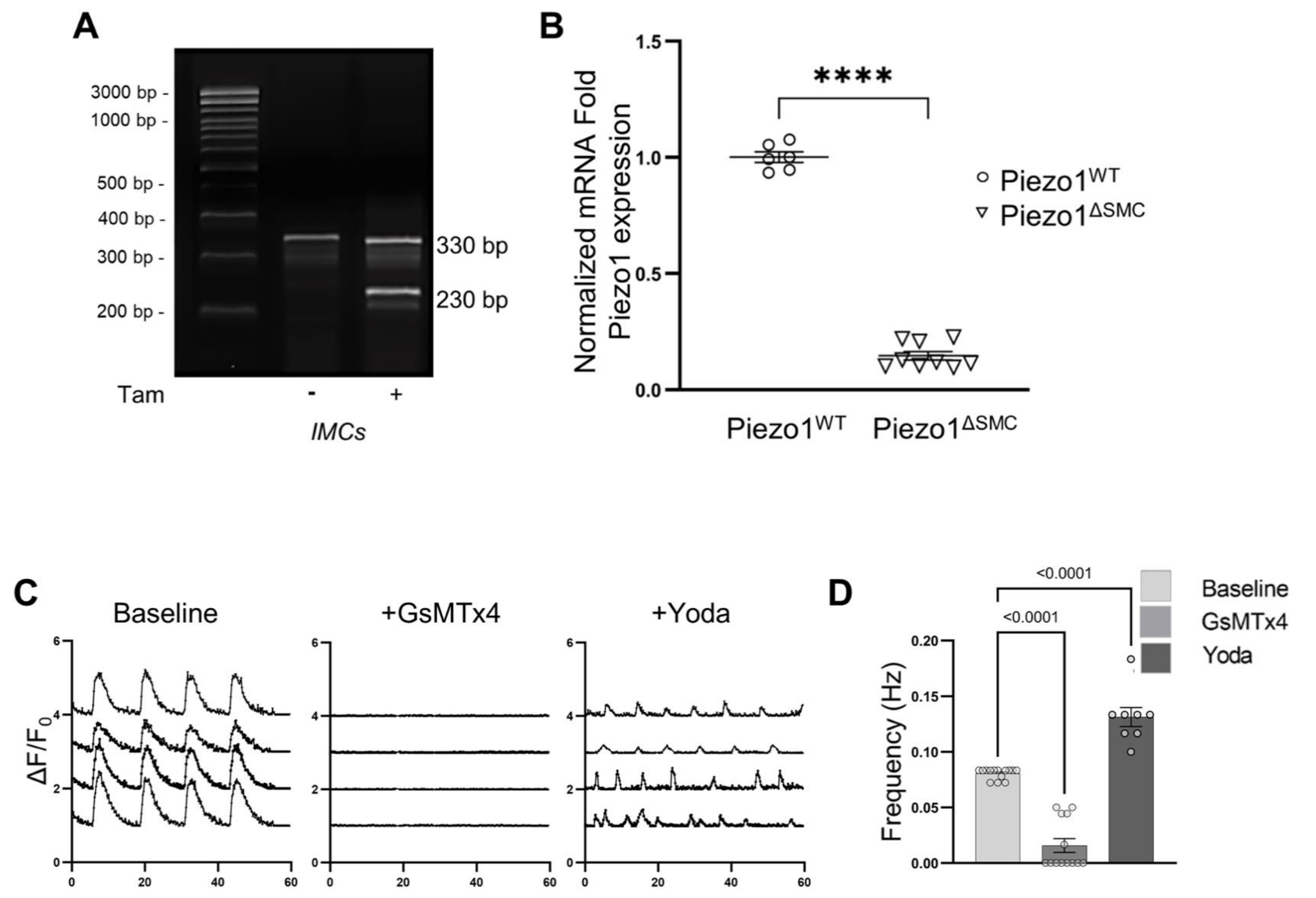

(A/B*)* *In vitro* confirmation of muscularis sample knockout efficiency as determined by PCR and qPCR of appropriate cleavage[30] of floxed Piezo1$^{\Delta SMC}$ treated with 4-OHT or vehicle control of 7–10-day-old Piezo1$^{\Delta SMC}$ samples. The qPCR results were calculated using the ΔΔCT method relative to Gapdh expression. **(C)** Representative GCaMP6f Ca^{2+} tracings of human IMCs extracted from fetal intestinal muscularis samples (17 weeks gestational age) subjected to GsMTx4 (20μM) and Yoda1 (5μM), with **(D)** frequency differences (n>3 measurements/sample, n>5 biological specimens per group). One-way ANOVA with Dunnett's correction was performed to compare it to the baseline. Data displayed as mean +/- SEM. p-value displayed above comparisons, with significance marked as $p < 0.05$.

Supplementary Figure 3: Quantification of SMCs by layer.

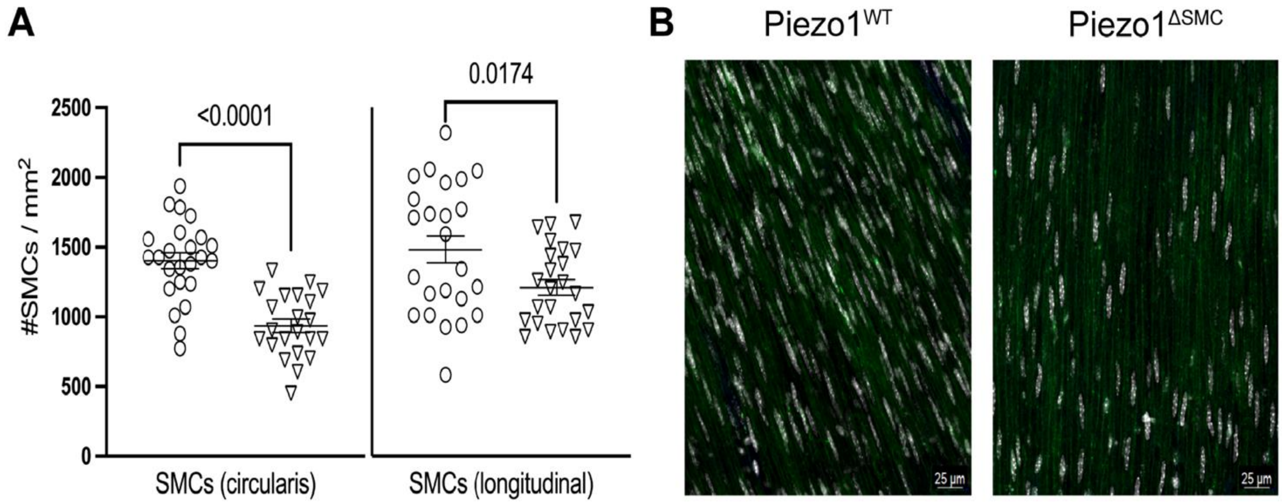

(A) The SMC density (SMCs per mm2) in separate layers (circular and longitudinal) using distal small bowel segments taken from 7-9 week-old mice (following Tam administration at 4-6 weeks old) with all assessments performed age-matched in both groups. (B) Representative WM preparations of distal small bowel of Piezo1^{WT} (left) and Piezo1$^{\Delta SMC}$ (right) mice at post-Tam treatment showing SMCs (pseudo-white). Scale bars are 25 μm.

Supplementary Figure 4: Whole-mount imaging of cellular components of the muscularis.

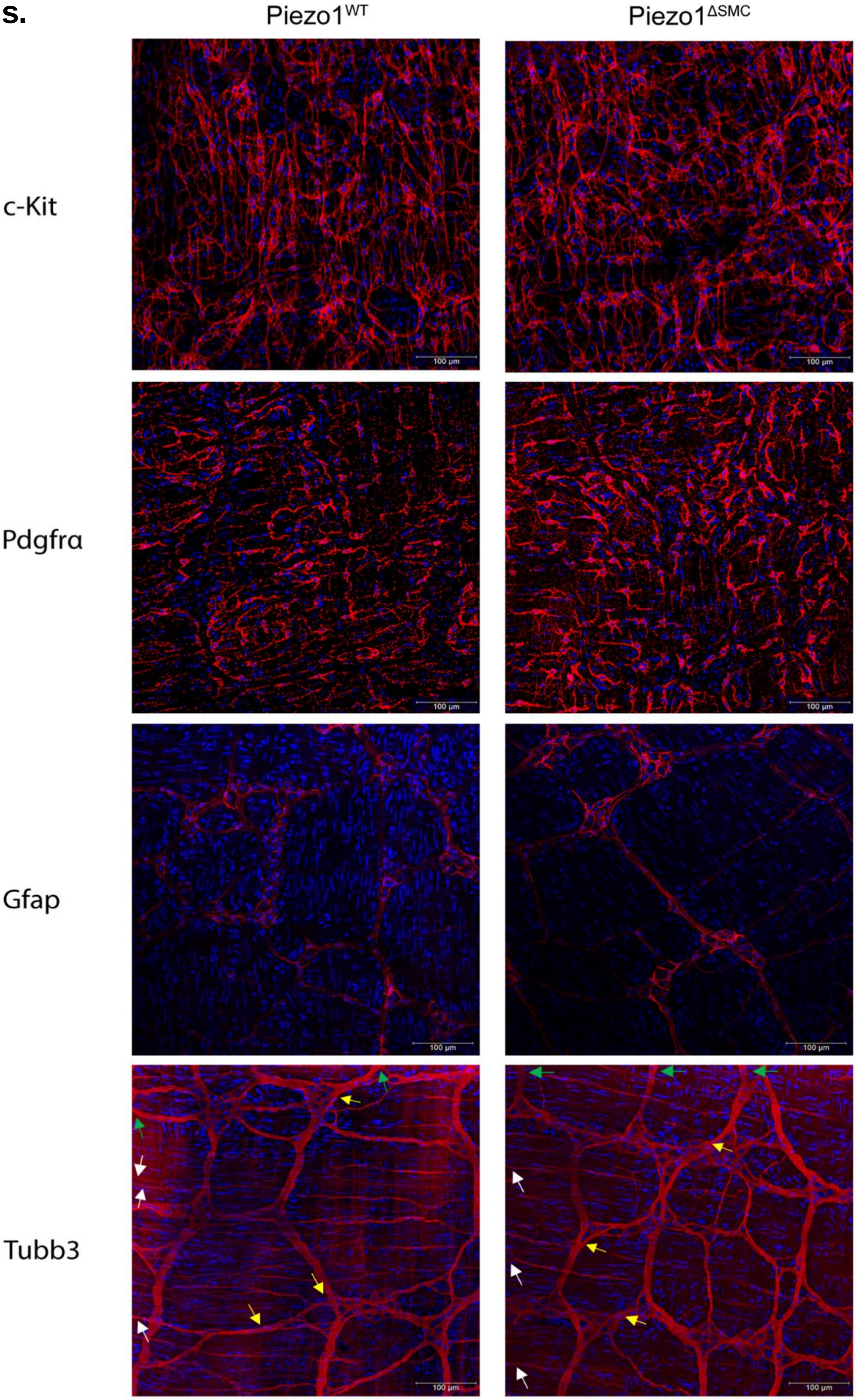

WM longitudinal samples of distal small bowel of Piezo1^{WT} (left) and Piezo1$^{\Delta SMC}$ (right) mice at 21 days post-Tam treatment (7-9 weeks) stained with DAPI, anti-c-Kit (ICC), Pdgfrα (Pdgfrα+), Gfap (Glial cells), and Tubb3 (neuronal cells, bundles, and fibers) antibodies. Tubb3 staining includes arrows to differentiate neuronal bundles (green), fibers (pseudo-white), and cells (yellow). Scale bars are 100μm.

Supplementary Figure 5: . SMC-specific deletion of Piezo1 alters related Ca^{2+} ion channel expression within the muscularis.

A

ΔCt		
**Gene**	$Piezo1^{WT}$	$Piezo1^{\Delta SMC}$
**Ano1**	14.332482	10.570477
**Orai1**	11.445477	14.527725
**Orai3**	10.164353	11.331921
**Trpc4**	18.452350	19.796135
**Cav1.2**	14.263201	13.088020

B **Ano1**

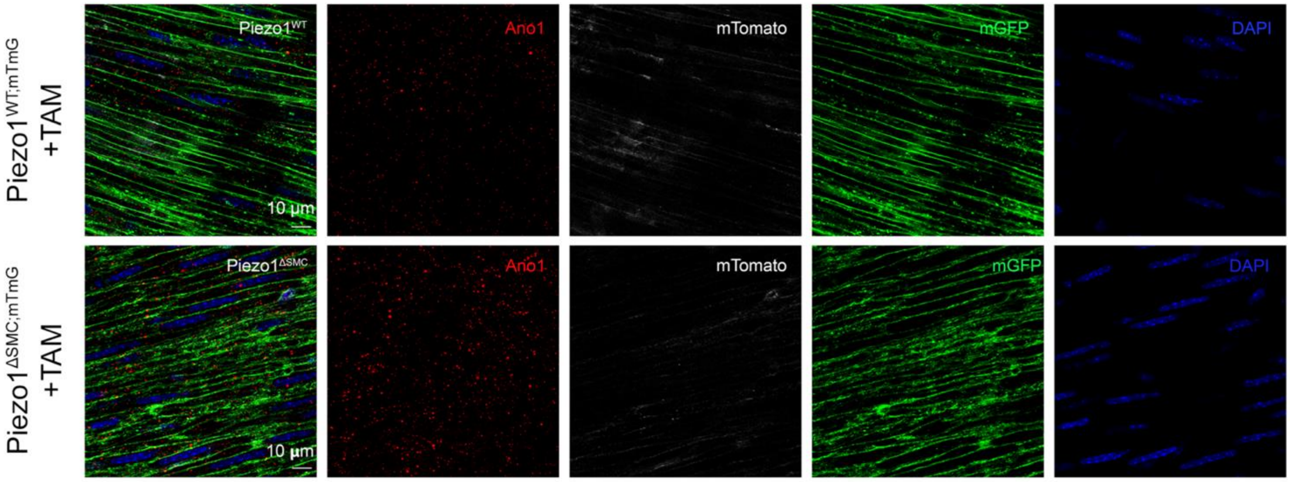

C **Orai1**

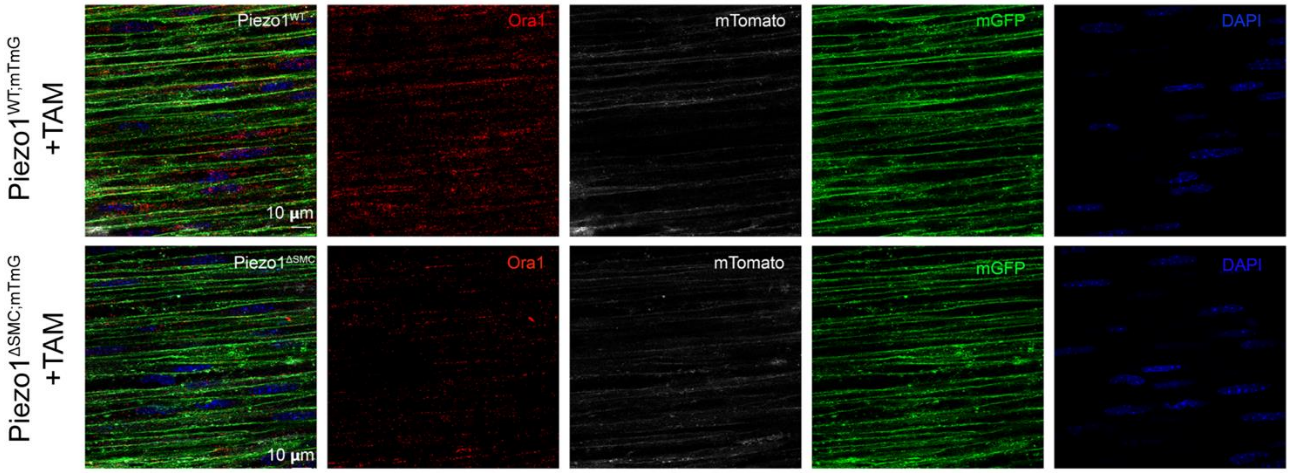

Distal small bowel samples were isolated from 4- to 6-week-old $Piezo1^{WT;mTmG}$ and $Piezo1^{\Delta SMC;mTmG}$ mice 21d post-Tam were **(A)** quantified for relative expression of related ion channels via qPCR (ΔCT values) shown for final graph in **Fig.5A**; with confocal imaging of **(B)** Ano1 and **(C)** Orai 1**.** Green shows the membrane-GFP; pseudo-white is the membrane-Tdtomato from the mTmG reporter. All antibodies were obtained from Alamone Labs using WM preparations. Scale bars 10μm.

Supplementary Figure 6: SMC-specific deletion of Piezo1 alters related Ca^{2+} ion channel expression within the muscularis.

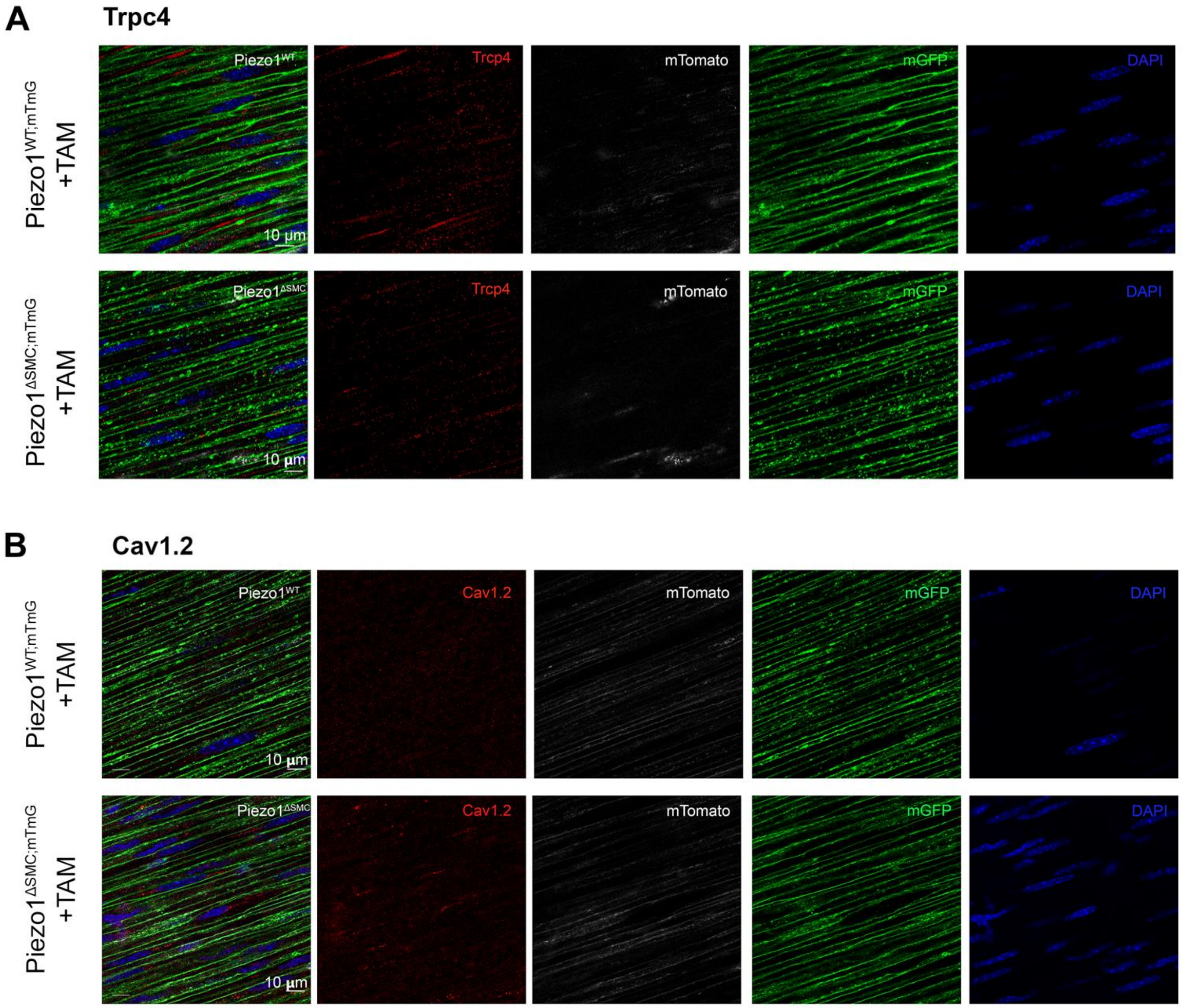

Distal small bowel samples were isolated from $Piezo1^{WT;mTmG}$ (upper) and $Piezo1^{\Delta SMC;mTmG}$ (lower) mice 21d post-Tam (7-9 weeks) were imaged with Leica Confocal microscope to assess IF staining of related ion channels. Green shows the membrane-GFP; pseudo-white is the membrane-Tdtomato from the mTmG reporter. Antibodies shown are **(A)** Trpc4 and **(B)** Cav1.2 with pseudo-red (Alamone) using WM preparations. Scale bars 10μm.

Supplementary Figure 7: Colocalization of Piezo1 with RyR and IP3R.

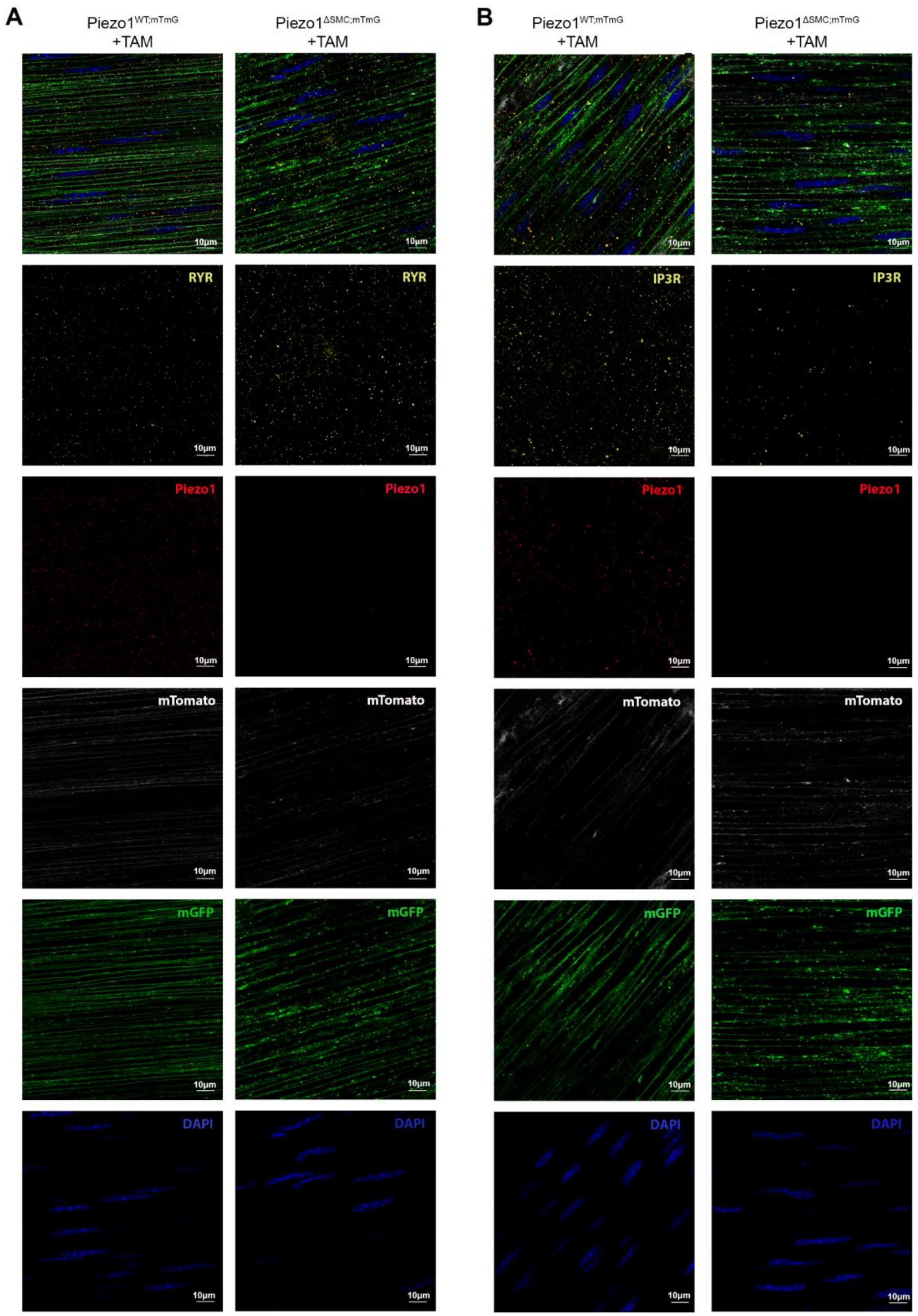

Leica Confocal SP8-STED microscope generated single-channel images of muscularis in **(A)** Figure 9A of anti-RyR (pseudo-yellow) and Piezo1 (pseudo-red), and green and pseudo-white from mTmG reporter from Piezo1$^{WT;mTmG}$ and Piezo1$^{\Delta SMC;mTmG}$ mice. **(B)** Figure 9B of anti-IP3R (pseudo-yellow) and Piezo1 (pseudo-red), and green and pseudo-white from mTmG reporter from Piezo1$^{WT;mTmG}$ and Piezo1$^{\Delta SMC;mTmG}$ mice. Scale bars 10μm.

Supplementary Figure 8: Nested analysis of RyR and IP3R positive clusters.

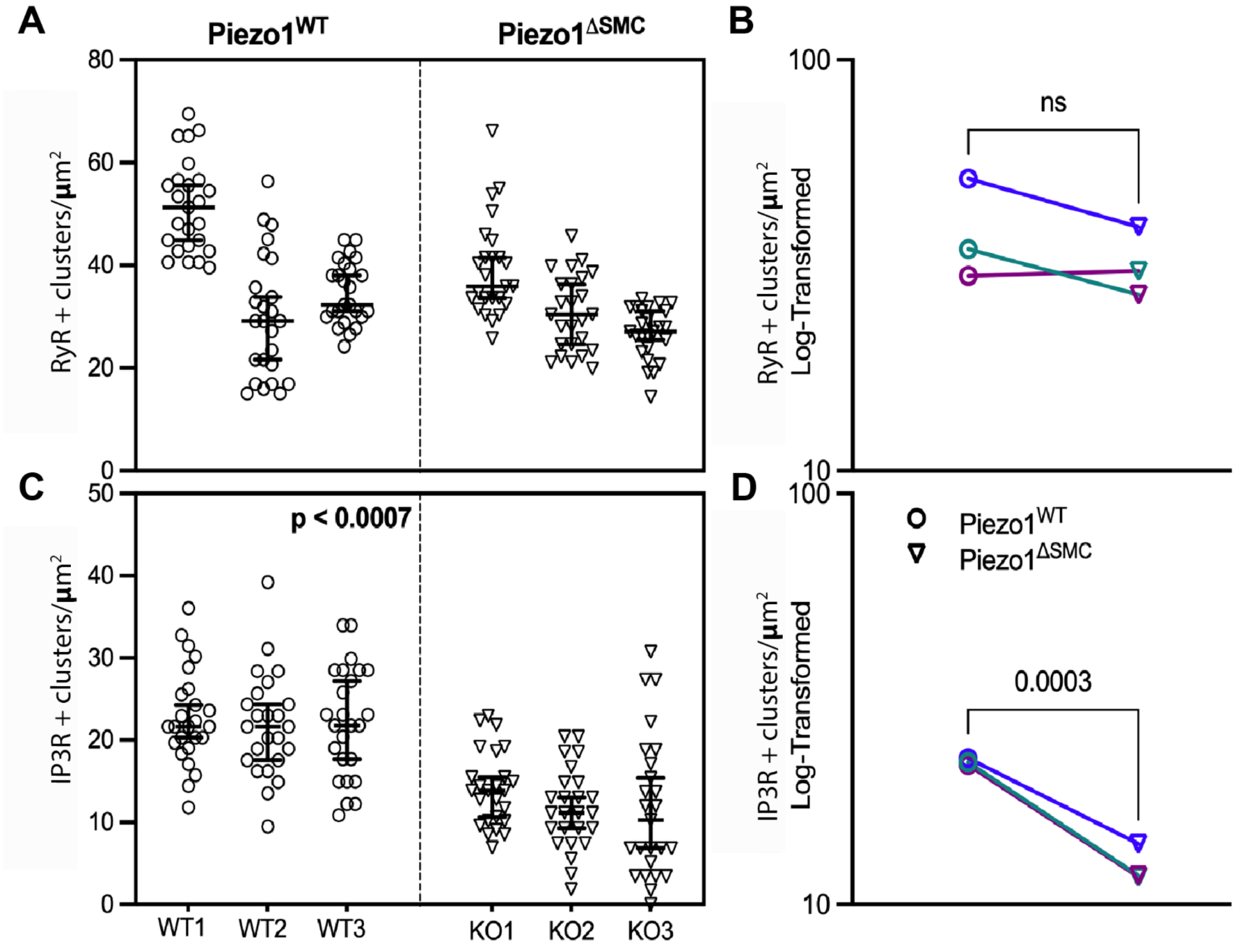

(A/C) To assess the impact of loss of Piezo1 on cells expressing RyR+(top) or IP3R+(bottom) signals or clusters, a nested t-test was conducted with each mouse designated as WT1,2,3 (wildtype) or KO1,2,3 (knockout). Total counts of specific protein clusters were obtained using using AIVIA software Pixel Classifier and Smart Segmentation tools to segment structures, distinguish them from the background, and generate 2D outlines. Total counts were then normalized by ROI area relative to sample proportion to account for batch and animal differences (n>25 ROIs per mouse, N=3 mice per group). **(B/D)** Log-transformed averages from each mouse displayed to illustrate directionality of general changes observed**.**

Supplementary Movie: Whole-mount imaging of the external muscularis. WM longitudinal sample of distal small bowel of Piezo1$^{WT;mTmG}$ a mouse treated with Tam. Leica Confocal SP8-STED microscope generated single-channel images. The z-stack movie between each image was 0.4um and goes from the longitudinal layer, which is membrane-associated GFP fluorescence (pseudo-green) positive and indicates Cre-activation. The adjacent myenteric plexus is membrane-associated, revealing Tdtomato fluorescence signals in most cells. The circular muscularis layer appears perpendicular to the longitudinal layer at a deeper level. Beyond the circular level are Tdtomato fluorescence cells in an irregular circular structure surrounding epithelial crypts that are not apparent.

Supplementary Tables

Supplementary Table 1: Antibodies				
**Antigen Target**	**Company**	**Catalog #, RRID**	**Dilution**	**Application**
Alexa Fluor Goat anti Rabbit 647	Thermo Fisher Scientific	A21246, AB_2535814	1:200	FFPE; WM
Alexa Fluor Donkey anti Mouse 647	Thermo Fisher Scientific	A-31571, AB_162542	1:200	WM
Alexa Fluor Donkey anti Mouse 488	Thermo Fisher Scientific	A21202, AB_141607	1:200	FFPE; WM
DyLight Goat anti Guinea Pig 800	Thermo Fisher Scientific	SA5-10100, AB_2556680	1:200	WM
Alexa Fluor Goat anti Mouse 790	Thermo Fisher Scientific	A11375, AB_2534146	1:200	WM
β-III Tubulin	Abcam	Ab78078, AB_2256751	1:500	WM
β-actin	BD Biosciences	612657, AB_399901	1:200	FFPE
CC3	Cell Signaling	9661S, AB_2341188	1:50; 1:400	FFPE; WM
c-Kit/CD117	R&D Systems	AF1356, AB_354750	1:100	WM
Pdgfra (D13C6)	Cell Signaling	5241, AB_10692773	1:1000	WM
Gfap	Abcam	ab7260, AB_305808	1:1000	WM
Piezo1	Alamone Labs	APC-087, AB_2756743	1:500	FF; WM
Piezo1	Proteintech	15939-1-AP, AB_2231460	1:1000	WB
Piezo2	Alamone Labs	APC-090, AB_2876842	1:100	WM
β-Tubulin	DSHB	E7, AB_528499	1:1000	WB
Cav1.2	Alomone Labs	ACC-003-GP, AB_11219156	1:500	WM
Orai1	Alomone Labs	ACC-062, AB_10918021	1:500	WM
Ano1	Alomone Labs	ACL-011, AB_10920576	1:200	WM
Trpc4	Alomone Labs	ACC-119, AB_234091	1:250	WM
RyR (F-1)	Santa Cruz	sc-376507, AB_11149759	1:100	WM
IP3R-3	BD Bioscience	610312, AB_397704	1:500	WM

Supplementary Table 2: Oligonucleotides		
**Common Name**	**PCR/qPCR**	**Reference/Thermo Fisher Taqman #**
Piezo1	PCR	**P1 F:** CTTGACCTGTCCCCTTCCCCATCAAG;<br>**P1 WT/fl R:** CAGTCACTGCTCTTAACCATTGAGCCATCTC;<br>**P1 KO R:** AGGTTGCAGGGTGGCATGGCTCTTTTT [30]
Piezo1	qPCR	Assay ID: Mm01241549_m1
Piezo2	qPCR	Assay ID: Mm01265861_m1
Atoh1	qPCR	Assay ID: Mm00476035_s1
Ano1	qPCR	Assay ID: Mm00724407_m1
Trpc4	qPCR	Assay ID: Mm00444280_m1
Orai1	qPCR	Assay ID: Mm00774349_m1
Orai3	qPCR	Assay ID: Mm01612888_m1
Cav1.2	qPCR	Assay ID: Mm01188822_m1
Gapdh	qPCR	Assay ID: Mm99999915_g1

Supplementary Table 3: Lentivirus		
**Common Name**	**Formal Name**	**Order Stock #**
gCAMP6f	Phage-RSV-gCAMP6f	Addgene #80315
mCherry	pLV-mcherry	Addgene # 36084
Scramble shRNA	TRIPZ Inducible Lentiviral shRNA Nonsilencing Control	Thermo Scientific #RHS4743

Supplementary Table 4: Murine lines			
**Common Name**	**Formal Name**	**RRID#**	**JAX Stock #**
Myh11-ERT2/Cre-	B6.FVB-Tg(Myh11-icre/ERT2)1Soff/J	IMSR_JAX:019079	#019079
Piezo1$^{fl/fl}$	B6.Cg-*Piezo1*$^{tm2.1Apat}$/J	IMSR_JAX:029213	#029213
mTmG	B6.129(Cg)-Gt(ROSA)26Sortm4(ACTB-tdTomato,-EGFP)Luo/J	IMSR_JAX:007676	#007676

Supplementary Table 5: Drugs

Modulator	Target Gene	Concentration	Company	Catalog #
**Yoda**	Piezo-1 Activator	5µM	Tocris	558638
**GSMTx4**	Piezo-1, Trpc Inhibitor	20µM; 1µM	MedChemExpress USA	HY-P1410A
**Verapamil**	L-type Ca Channel Blocker	10µM	Cytoskeleton	CY-SCV01
**Carbachol**	M3 agonist	10µM; 1µM	Sigma	51-83-2
**Nicardipine**	Ca Channel Blocker	10µM	Sigma	4527-84-3
**5-HT**	Serotonin activator	10µM	Sigma	50-67-9
**TTX**	Inhibits voltage-gated sodium channels	1µM	TOCRIS	1069
**ODQ**	Guanylyl cyclase Nitric oxide inhibitor	10µM	Cayman Chemical	81410
**L-NNA**	Nitric oxide synthase (NOS) inhibitor	100µM	Cayman Chemical	80220
