# Supplementary material for: Smooth muscle cell Piezo1 depletion results in impaired contractile properties in murine small bowel
Source: Commun Biol. 2025 Mar 17;8:448. doi: 10.1038/s42003-025-07697-6 (PMC11914552; doi:10.1038/s42003-025-07697-6)
Supplement: Supplementary file 2 — Description of Additional Supplementary Files [file 42003_2025_7697_MOESM2_ESM.docx]

**Description of Additional Supplementary Files**

File name: Supplementary Data

Description: The source data behind the graphs in the paper

File name: Supplementary Movie

Description: Whole-mount imaging of the external muscularis.
