# Supplementary material for: Smooth muscle cell Piezo1 depletion results in impaired contractile properties in murine small bowel
Source: Commun Biol. 2025 Mar 17;8:448. doi: 10.1038/s42003-025-07697-6 (PMC11914552; doi:10.1038/s42003-025-07697-6)
Supplement: Supplementary file 4 — Reporting Summary [file 42003_2025_7697_MOESM4_ESM.pdf]

nature portfolio

Corresponding author(s): Geoanna Bautista, MD
Martin G. Martin, MD

Last updated by author(s): Jan 18, 2025

Reporting Summary

Nature Portfolio wishes to improve the reproducibility of the work that we publish. This form provides structure for consistency and transparency in reporting. For further information on Nature Portfolio policies, see our Editorial Policies and the Editorial Policy Checklist.

Statistics

For all statistical analyses, confirm that the following items are present in the figure legend, table legend, main text, or Methods section.

n/a	Confirmed	
☐	☒	The exact sample size (*n*) for each experimental group/condition, given as a discrete number and unit of measurement
☐	☒	A statement on whether measurements were taken from distinct samples or whether the same sample was measured repeatedly
☐	☒	The statistical test(s) used AND whether they are one- or two-sided<br>*Only common tests should be described solely by name; describe more complex techniques in the Methods section.*
☐	☒	A description of all covariates tested
☐	☒	A description of any assumptions or corrections, such as tests of normality and adjustment for multiple comparisons
☐	☒	A full description of the statistical parameters including central tendency (e.g. means) or other basic estimates (e.g. regression coefficient) AND variation (e.g. standard deviation) or associated estimates of uncertainty (e.g. confidence intervals)
☐	☒	For null hypothesis testing, the test statistic (e.g. *F*, *t*, *r*) with confidence intervals, effect sizes, degrees of freedom and *P* value noted<br>*Give P values as exact values whenever suitable.*
☒	☐	For Bayesian analysis, information on the choice of priors and Markov chain Monte Carlo settings
☒	☐	For hierarchical and complex designs, identification of the appropriate level for tests and full reporting of outcomes
☐	☒	Estimates of effect sizes (e.g. Cohen's *d*, Pearson's *r*), indicating how they were calculated

Our web collection on statistics for biologists contains articles on many of the points above.

Software and code

Policy information about availability of computer code

Data collection	Calcium influx signaling data and imaging from in vitro experiments were collected using ZEISS ZEN Microscopy Software. LabChart 8 (Pro version, ADinstruments) was utilized for data acquisition, recording and analysis for isometric contractility assessments. Both are commercial software products that can be purchased.
Data analysis	Calcium data analysis was carried out using Zen3.3 blue addition and custom codes using the Matlab. All statistical analyses were performed using Graph Pad Prism version 10.0.0 for Windows (Graph Pad Software, Boston, Massachusetts USA, www.graphpad.com). All custom codes developed to aid in analyses will be submitted with manuscript files. Cwodes used related to any quantification have been uploaded to a repository and link /citation included in methods of the manuscript.

For manuscripts utilizing custom algorithms or software that are central to the research but not yet described in published literature, software must be made available to editors and reviewers. We strongly encourage code deposition in a community repository (e.g. GitHub). See the Nature Portfolio guidelines for submitting code & software for further information.

April 2023

Data

Policy information about availability of data

All manuscripts must include a data availability statement. This statement should provide the following information, where applicable:

- Accession codes, unique identifiers, or web links for publicly available datasets
- A description of any restrictions on data availability
- For clinical datasets or third party data, please ensure that the statement adheres to our policy

All data used to generate the figures have been combined and separated by figure. This will be accessible via linked supplementary files and/or appropriate data repository

Research involving human participants, their data, or biological material

Policy information about studies with human participants or human data. See also policy information about sex, gender (identity/presentation), and sexual orientation and race, ethnicity and racism.

Reporting on sex and gender	N/A
Reporting on race, ethnicity, or other socially relevant groupings	N/A
Population characteristics	N/A
Recruitment	N/A
Ethics oversight	N/A

Note that full information on the approval of the study protocol must also be provided in the manuscript.

Field-specific reporting

Please select the one below that is the best fit for your research. If you are not sure, read the appropriate sections before making your selection.

☒ Life sciences ☐ Behavioural & social sciences ☐ Ecological, evolutionary & environmental sciences

For a reference copy of the document with all sections, see nature.com/documents/nr-reporting-summary-flat.pdf

Life sciences study design

All studies must disclose on these points even when the disclosure is negative.

Sample size	We determined sample size per each experiment type using a sample size that provided 80% power to detect effect sizes of at least 2.03, assuming a two-sample t-test with a 0.05 two-sided level of significance
Data exclusions	Predetermined criteria removal of all data generated from an entire mouse if PCR/qPCR determined cre-induced Piezo1 deletion inadequate (<20% reduction compared to wildtype) or if animal was sick, which may impact specific outcomes measured; also if deviation n in technical/ experimental process was found to cause large variation in data analysis (need to recalibrate machinery, chemicals not dissolving well, unintended fluctuations in pH or temperature) and resulted in unexpected results.
Replication	All experiments were performed in at least triplicates, on several animals from different litters to control for litter effects
Randomization	Randomization was not possible as genotyping needed to be done in order to confirm efficiency of knockout and subsequent studies. However, both littermate controls and multiple litters were used to adequately represent data.
Blinding	For histological and immuno-flourescence data - measurements performed by at least two different investigators and measured for congruence.

Reporting for specific materials, systems and methods

We require information from authors about some types of materials, experimental systems and methods used in many studies. Here, indicate whether each material, system or method listed is relevant to your study. If you are not sure if a list item applies to your research, read the appropriate section before selecting a response.

Materials & experimental systems

n/a	Involved in the study
☐	☒ Antibodies
☒	☐ Eukaryotic cell lines
☒	☐ Palaeontology and archaeology
☐	☒ Animals and other organisms
☒	☐ Clinical data
☒	☐ Dual use research of concern
☒	☐ Plants

Methods

n/a	Involved in the study
☒	☐ ChIP-seq
☒	☐ Flow cytometry
☒	☐ MRI-based neuroimaging

Antibodies

Antibodies used	All antibodies used with their RRIDs and catalog numbers are available in the methods section in Table 1
Validation	We tested that the Piezol antibody (Alamone Labs) that was validated with our transgenic ere mouse model confirming absence of Piezol in Myhll + when activated. All other commercial antibodies have been verified by others and listed in table l.

Animals and other research organisms

Policy information about studies involving animals; ARRIVE guidelines recommended for reporting animal research, and Sex and Gender in Research

Laboratory animals	Myhll-ERT2/Cre- B6.FVB-Tg(Myhll-icre/ERT2)1Soff/J # 019079<br>Piezolfl/fl B6.Cg-Piezoltm2.1Apat/J #029213<br>mTmG B6.129(Cg)-Gt(ROSA)26Sortm4(ACTB-tdTomato,-EGFP)Luo/J #007676<br>Rosa26 B6(129S4)-Gt(ROSA)26Sortml.l(CAG-tdTomato/GCamp6F)Mdcah/J #031968
Wild animals	N/A
Reporting on sex	An inherent limitation of the mouse model used in this study includes excluding female mice in KO studies because Cre is only carried on the Y chromosome in the Myhll-CreERT model. This is a widely used model and what was available at the time of this study.
Field-collected samples	N/A
Ethics oversight	All experiments were approved by the UCLA Institutional Animal Care and Use Committee (IACUC) (Protocol #).<br>All human intestinal samples were obtained from deidentified and discarded fetal tissue specimens following pathology evaluation by the UCLA translational pathology core through an approved IRB approved by the UCLA Institutional Review Board (IRB #11-002504).

Note that full information on the approval of the study protocol must also be provided in the manuscript.

Plants

Seed stocks	N/A
Novel plant genotypes	N/A
Authentication	N/A
